# Comprehensive analysis of miRNA profiles reveals the role of *Schistosoma japonicum* miRNAs at different developmental stages

**DOI:** 10.1186/s13567-019-0642-2

**Published:** 2019-04-04

**Authors:** Jie Yu, Ying Yu, Qing Li, Muxin Chen, Haimo Shen, RuiXiang Zhang, Mingxin Song, Wei Hu

**Affiliations:** 1 College of Veterinary Medicine, Northeast Agricultural University, Harbin, 150030 China; 2 State Key Laboratory of Genetic Engineering, Ministry of Education Key Laboratory of Contemporary Anthropology, Collaborative Innovation Center for Genetics and Development, School of Life Sciences, Fudan University, Shanghai, 200438 China; 3 Joint Research Laboratory of Genetics and Ecology on Parasite-host Interaction, Chinese Center for Disease Control and Prevention & Fudan University, WHO Collaborating Centre for Tropical Diseases, National Center for International Research on Tropical Diseases, Key Laboratory of Parasite and Vector Biology Ministry of Health, National Institute of Parasitic Diseases, Shanghai, 200025 China; 4 Heilongjiang Key Laboratory for Zoonosis, Harbin, 150030 China

## Abstract

Schistosomiasis is an important zoonotic disease affecting up to 40 kinds of animals and 250 million people. It has been reported that the miRNAs play a role in the metabolism, differentiation, development and reproduction in many organisms. However, the roles of miRNAs regulating the development, maturation and production in schistosome in both females and males remains unclear. Here we present the dynamic transcriptome analysis of all 79 known *Schistosoma japonicum* miRNAs from pairing to production, including 14 days post-infection (dpi), 16, 18, 20, 22, 24, 26, 28 dpi female and male, by small RNA sequencing. The miRNA expression profiles showed time-related characteristics in male and female from paring to production, which could be clustered into three patterns, characterized by pairing stage highly expressed (cluster 1), maturating stage highly expressed (cluster 2), and egg producing stage highly expressed (cluster 3). The enrichment of miRNA cluster targeted genes in female and male were distinctly different. Network analysis of miRNAs and their target regulation showed that cluster 1 had 15 miRNAs involved in the regulation of interaction, communication, immune response in female–male and parasite–host. The other 11 miRNAs were involved in gender differentiation and the meiotic cell cycle process. In cluster 2, 11 miRNAs were involved in development and sexual maturation. In cluster 3, 45 miRNAs possibly regulate metabolism and synthesis of the substance for egg production. Analysis of the miRNA regulation network would contribute to understanding the molecular mechanism in *S. japonicum* development and egg production.

**Electronic supplementary material:**

The online version of this article (10.1186/s13567-019-0642-2) contains supplementary material, which is available to authorized users.

## Introduction

Schistosomiasis, a zoonotic parasitic disease, is caused by blood flukes of *Schistosoma* spp. Schistosomiasis has a wide range of definitive hosts, not only more than 40 kinds of animals including cattle, goats, sheep, camels, canine, swine, equine, equus ferus × asinus, macaca, felidae and rodents and avian [1–4], but also affecting 258 million people in 76 countries in tropical and subtropical regions. *Schistosoma japonicum* (*S. japonicum*) is prevalent in Asia, primarily China and the Philippines [5, 6]. After infection, schistosomula migrate into the host’s bloodstream, where they develop into sexual maturation and lay eggs. Dioecy is a unique characteristic of schistosomes, in which the adult male and female live in a copula, while the female does not mature without a male [7–9]. Each paired female *S. japonicum* can deposit 1500–3000 eggs per day, which is about 5 to 10 times more than that of *Schistosoma mansoni* (*S. mansoni*). Thus, *S. japonicum* causes more severe morbidity than other schistosome species [9, 10]. Schistosome sex differentiation, maturation and egg production are the key events contributing to consequent morbidity and transmission. Understanding the molecular mechanisms of these processes is extremely urgent in current investigations on trematodes. MicroRNAs (miRNAs) are a class of small non-coding RNAs (~22 nucleotides in length), which play a crucial role in regulation of gene expression by binding to target messenger RNA (mRNA) and triggering translation repression or mRNA degradation [11, 12].

MicroRNAs have been reported to be involved in post-transcriptional regulation of gene expression during development, differentiation, proliferation, death and metabolism in a variety of organisms [13]. To date an increasing number of schistosome miRNAs from various development stages have been identified with transcriptome analysis and deep-sequencing technique in *S. japonicum* [14–18] and *S. mansoni* [19–21]. miRNA and endogenous siRNA expression in *S. japonicum* were profiled at the stages of cercariae, lung-stage schistosomula, eggs sequestered in tissues, adults of both sexes by deep sequencing and qRT-PCR methods, which provided a broader view of small RNAs of the parasite [22, 23]. A study on sex differential expression of miRNAs in *S. mansoni* found that 10 of 13 microRNAs were more abundant in females than in males, exhibiting sex-biased expression patterns, and thus pointing towards a plausible involvement of miRNAs in the sex differentiation and maintenance [21]. Sun et al. investigated the differential expression profiles of *S. japonicum* miRNAs in mature and immature worms, and they also analyzed miRNAs’ target genes. The results showed that more genes and metabolic pathways were regulated by miRNAs in paired mature females than in unpaired immature females [17]. Sequencing of small RNAs from 16, 22 and 28 dpi *S. japonicum* showed that 18 miRNAs are expressed differently between male and female, and that by knocking down the expression of female enriched miRNAs sja-bantam and sja-miR-31, morphological alterations were observed in female ovaries [18]. Recently, Protasio et al. reported that a novel *S. mansoni* miRNA family (sma-miR-277/4989) might play a dominant role in post-translational regulation in the sexual development of female schistosomes [24]. Wang’s report [25] showed that *S. japonicum* pairing began at as early as 16 dpi. At 20 dpi, oogonium and spermatogonium were initially differentiated and mature vitelline cells firstly appeared in females. At 24 dpi, mature germ cells and vitelline glands had appeared in most of females. Meanwhile, at 20–22 dpi and 24–26 dpi, thousands of transcripts were down regulated in female worms. Therefore, 14–16 dpi, 20–24 dpi, 24–26 dpi were crucial stages for *S. japonicum* pairing, development and maturation [25]. However, all known 79 *S. japonicum* miRNAs expression profiles and the role of miRNAs during those developmental stages (14–16 dpi, 20–24 dpi, 24–26 dpi) are currently unknown.

To gain insight into the role of *S. japonicum* miRNAs in the developmental process, particularly in gender differentiation and maturation, we utilized high-throughput small RNA sequencing to quantify the dynamic expression profile of miRNAs at 14, 16, 18, 20, 22, 24, 26, 28 dpi *S. japonicum* worms. Our results demonstrated that 79 known miRNAs had time-related characteristics in male and female. Enrichments of each miRNA cluster targets in female and male were distinctly different. Network analysis showed different miRNAs might play critical roles in the pairing, developmental and egg production stages. Our results also showed that different transferase activities were enriched in F-cluster 3, M-cluster 1 and M-cluster 3, which suggested that miRNAs might participate in the post-translational modification during different developmental stages. Global view and specific understanding of miRNA roles would contribute to study the molecular mechanism of *S. japonicum* developmental and maturation events.

## Materials and methods

### Animals, parasite and worm collection

*Schistosoma japonicum* was harvested by hepatic-portal perfusion from mice (C57BL/6, female, 5–6 weeks) infected with cercariae released from *Oncomelania hupensis* snails (Anhui isolate). The infected snails were provided by the National Institute of Parasitic Diseases, Chinese Center for Disease Control and Prevention, Shanghai. Worms were collected at 8 time points on every other day from 14 dpi through to 28 dpi. The sampled worms were washed with sterile DMEM (Dulbecco’s Modified Eagle’s medium) three times. Male and female worms were separated under light microscopy and stored in RNAlater stabilization solution (Invitrogen, USA) at −80 °C before use. Assays were processed with worms from one mouse representing one biological replicate, all assays in our study had three biological replicates.

### RNA extraction, small RNA library construction and sequencing

*Schistosoma japonicum* total RNA was isolated using Qiagen miRNeasy Mini Kit (Valencia, CA, USA) according to the manufacturer’s instruction. The concentration and purity were evaluated spectrophotometrically using an Agilent 2100 Bioanalyzer (Agilent Technologies, Palo Alto, CA, USA) and a NanoDrop ND2000 spectrophotometer at 260 nm and 280 nm. The small RNA library was constructed as previously described [26–28]. Briefly, 10 µg total RNA was separated by electrophoresis on a Novex 15% TBE-Urea gel (Invitrogen Co. Ltd) and RNA in length of 18–30 nt was purified and ligated to 3′ and 5′ adapters. Then for reverse transcription, we used a RT-PCR kit (Invitrogen Co. Ltd) to produce complementary DNA (cDNA). Finally, the cDNA was amplified using adapter primers, the products were purified using a 6% TBE PAGE gel (Invitrogen Co. Ltd) and sequenced with Illumina Genome Analyzer (Illumina HiSeq 2000) at the BGI (Beijing Genomics Institute, Shenzhen, China).

### Analysis of sequencing data

The quality of the RNA-Seq data was first examined using the package FastQC [29]. After a trimming process using Trimmomatic [30] adapters, PCR sequences and bases with low quality in the reads were clipped. A sequence read was discarded if it was below 17 bases in length. Using miRDeep v.2.0 [31], reads were aligned to the reference *S. japonicum* genome [32] from wormbase and known miRNAs database (downloaded from miRBase v20 [33]). Candidate miRNAs were identified with the miRDeep2 module using the core algorithm in RNAfold tool [31] to predict RNA secondary structures and evaluate the structure and signature of each putative miRNA precursor. Predicted miRNAs with significant Rand fold α level (*P* < 0.05) and miRDeep2 score (≥ 5) were considered as candidate miRNAs. Normalized expression profile (log scale) was generated by the quantifier module in miRDeep2 v.2.0.

### Validation of miRNAs expression by qRT-PCR

#### RNA extraction, and reverse transcription

Total RNA of *S. japonicum* male or female worms were isolated using Trizol reagent (Life Technologies, USA). Genome DNA was removed from total RNA with the DNase I (Takara, Japan). For polyadenylation and reverse transcription, poly-A tails were added to total RNA (1 μg) uncontaining genome DNA with ATP by *Escherichia coli* (*E. coli*) poly (A) polymerase using miScript PCR Starter Kit (Qiagen, Germany). Controls without reverse transcriptase were included for each sample.

#### Real-time PCR amplification of miRNAs

We applied poly-A quantitative reverse transcription PCR (qRT-PCR) method [34] to amplify miRNAs. For each PCR amplification, approximately 50 ng cDNA was mixed with 10 μM forward primer and 10 μM reverse primer (universal primer) (Qiagen, Germany), 10 μL 2× QuantiTect SYBR Green qPCR master mix (Qiagen, Germany) and sterile water in a final volume of 20 μL. Cycling conditions were: 95 °C for 5 min, followed by 45 cycles of 95 °C for 15 s, 55 ± 2 °C for 30 s and 72 °C for 30 s. Dissociation curve analysis was carried out at the end of each PCR run to verify amplification specificity for each miRNA. All reactions were run in triplicate, each sample has three technical parallel replicates and each group has three biological replicates. Sj-U6 snRNA was used in the qPCR reaction as an endogenous reference with forward primer 5′-GCAAGGATGACACGCAAAT-3′ and reverse primer 5′-ATGGAACGCTTCACGAAT-3′ [35]. Data analysis for Real-time PCR amplification efficiency and for quantifying relative miRNA expression were calculated using the 2^−ΔΔCT^ method [36].

### MiRNA target prediction

We used two software packages, RNAhybrid [37] and miRanda [38], to predict miRNA targets. In this study, we predicted the potential miRNA target sites not only with 3′ untranslated region (3′ UTR), but also with coding sequence (CDS) and 5′ UTR as the Ref. [11, 39, 40]. The mRNAs were based off the latest dataset of *S. japonicum* whole transcriptome (GenBank accession no. PRJNA343582). The cutoff was set: RNAhybrid with e-value ≤ 1e−20, minimum free energy (MFE) ≤ −20 kcal/mol. miRanda was used with a cutoff score of ≥ 140, energy cutoff ≤  −20 kcal/mol, gap opening = − 9.0 and gap extension = −4.0. Target score (i.e. the overall score of a miRNA-3′ UTRs pair in case that multiple binding sites occur) is the sum of scores for all binding sites. The miRNA potential targeted genes predicted by both tools were selected for the following network analysis.

### Luciferase system reporter assay and cell culture

MiRNA mimics and NC mimics (2′-*O*-methyl oligonucleotides) were synthesized chemically by Genepharma (Shanghai, China), the miRNA target binding site fragments were inserted into the multiple cloning sites of pmirGLO vector dual-luciferase report system (Promega, USA). HEK293T cells were cultured in 10% fetal bovine serum in DMEM (Gibco, USA), 800 nanograms of recombinant plasmid and 20 pmol miRNA mimics (GenePharma, China) were co-transfected into HEK293T cells by Lipofectamine 2000 (Invitrogen, USA) in 24-well plates. Firefly and renilla luciferase activity were measured after 24 h transfection by the dual luciferase assay system (Promega, USA).

### MiRNA cluster and GO analysis

The miRNA clusters were processed by MeV 4.9 software based on their normalized expression level. MiRNAs expression profile in female were patterned into three clusters: F-cluster 1, F-cluster 2 and F-cluster 3. MiRNAs expression profile in male also were patterned into three clusters: M-cluster 1, M-cluster 2 and M-cluster 3.

GO IDs of target genes were obtained with the BioProject no. PRJNA343582. The Gene Ontology (GO) enrichment analysis of the 6 miRNA clusters were processed by Blast2GO [41] (v4.2). All potential targets from each miRNA cluster in female and male were pooled together and enrichment performed for male and female separately.

### MiRNAs network analysis

Firstly, we sorted out GO terms enriched in female and male clusters, respectively. Secondly, we listed the targets enriched in each GO enrichment. Thirdly, we sorted out the miRNAs regulating those targets (from RNAhybrid and miRanda prediction) in each GO enrichment in female and male cluster. Lastly, Network was used to show the relationship between enrichment and miRNAs in cytoscape3.6.0 [42].

## Results

### Characteristics of *S. japonicum* small RNAs sequencing

**Figure 1:**
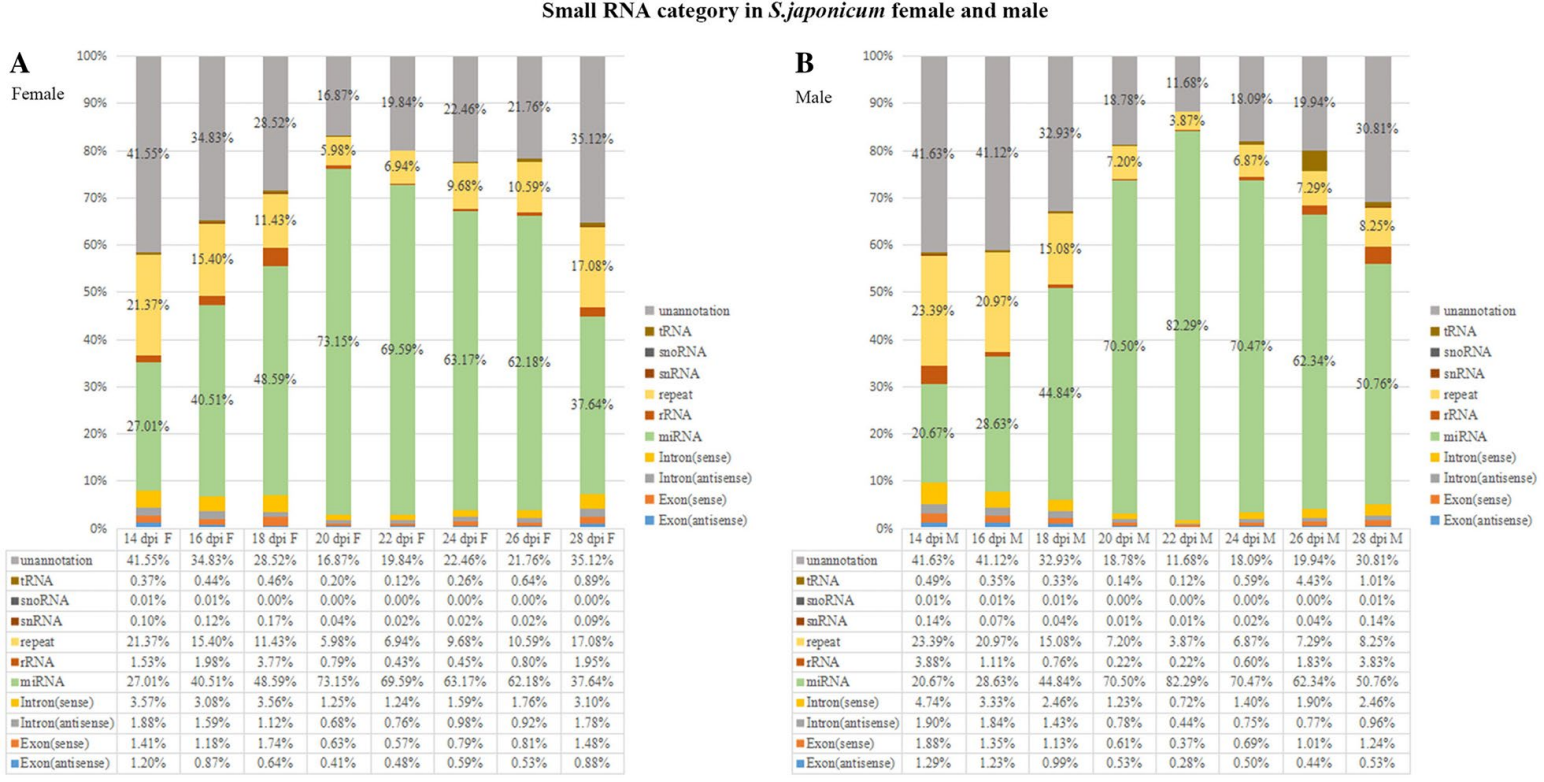
**Characterization of** ***S. japonicum*** **small RNA sequencing.** The percentages of miRNAs, rRNA, tRNA, snRNA, snoRNA, repeat, intron (sense), intron (antisense), exon (sense), exon (antisense), unannotated sequences in small RNA sequencing at eight developmental stages (14 dpi, 16 dpi, 18 dpi, 20 dpi, 22 dpi, 24 dpi, 26 dpi, 28 dpi). **A** Female, **B** male. rRNA: ribosomal RNA; tRNA: transfer RNA; snRNA: small nuclear RNA; snoRNA: small nucleolar RNAs.

To identify and profile *S. japonicum* miRNAs expression from pairing, maturation to egg production, we deep sequenced small RNAs in 48 *S. japonicum* samples (two genders, eight developmental stages, in triplicate). Flow charts of sequencing data analysis are shown in Additional file 1. Results (Figure 1, Additional file 2) showed the highest proportion of total small RNAs were miRNAs (~70%) at 20–26 dpi. The miRNA proportion was around 30–50% at 14–18 dpi and 28 dpi. Other small RNAs were composed by repeat sequences (3.87–23.39%), unannotated sequences (11.68–41.63%), intron (sense) (1.23–4.74%), intron (antisense) (0.44–1.90%), exon (sense) (0.37–1.88%), exon (antisense) (0.28–1.29%), rRNA (0.12–4.43%), tRNA (0.22–3.83%), snRNA (< 0.01%), snoRNA (< 0.01%) (Figure 1). The unannotated sequences might be endogenous siRNAs (small interfering RNAs) derived from the natural antisense transcripts (NAT) [22]. The ratios of small RNAs mapping to the genome were around 75%, ratios of mapping to miRNAs database were around 60% (Additional file 3A). Length of small RNAs were mainly between 20 and 23 nt (Additional file 3B). Our study detected all the 79 known *S. japonicum* miRNAs (miRBase 20.0 dataset) expression profiles from 14 to 28 dpi in different genders, as well as predicted 108 candidate miRNAs (Additional file 4) from unannotated sequences, in which two candidate miRNAs had the homologues with *S. mansoni* miRNAs (Additional file 4). The 108 candidate miRNAs still needed to be validated with experiments, e.g.: northern blot, stem-loop qRT-PCR [23, 35, 43].

### Dynamic profiles of *S. japonicum* miRNAs in female and male

**Figure 2:**
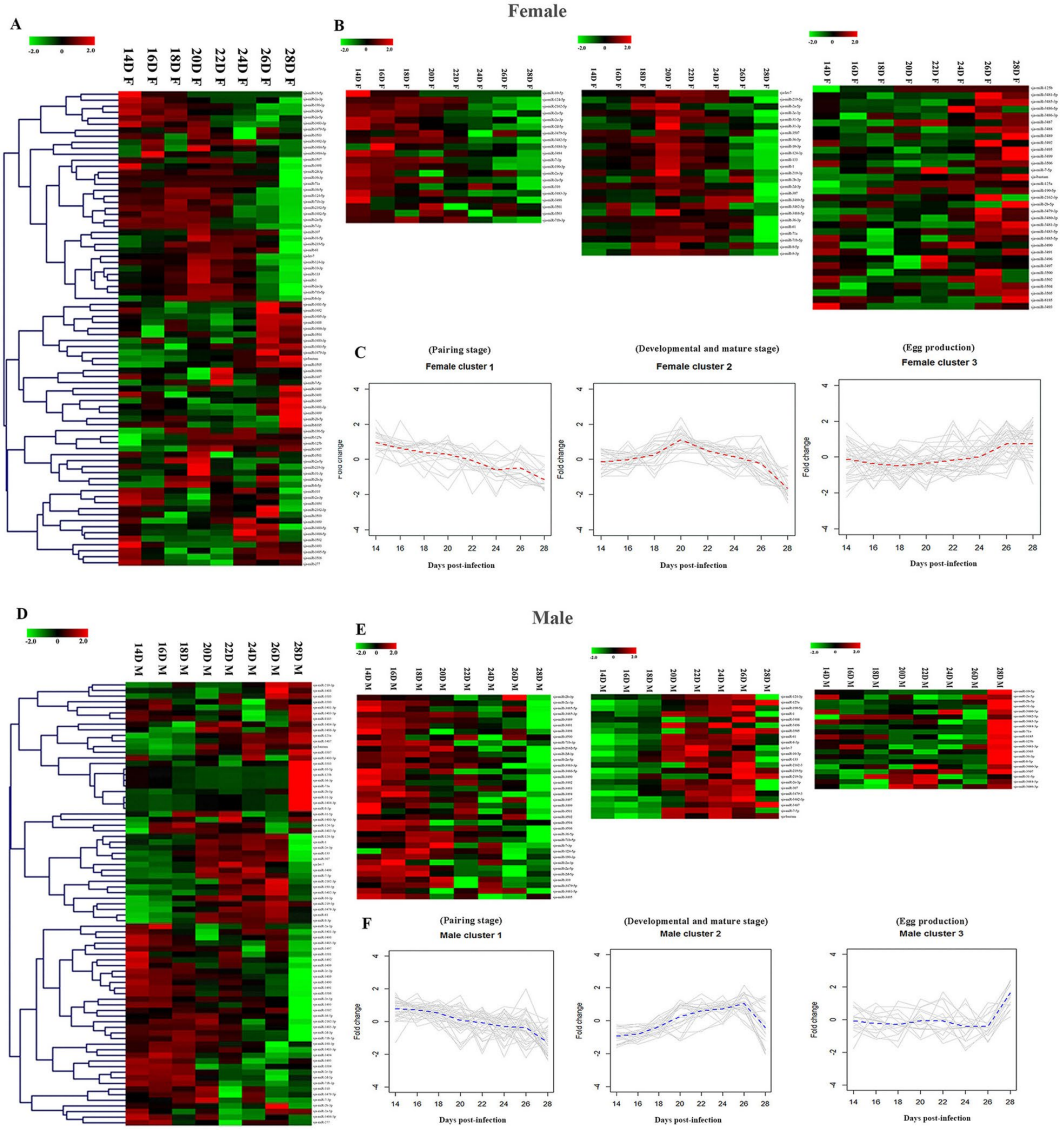
**Heatmap of 79** ***S. japonicum*** **known miRNAs expression profile.** MiRNAs were significantly high expression (red) or low expression (green). **A** Heatmap of 79 *S. japonicum* known miRNAs expression profile in female; **B** Three clusters of 79 *S. japonicum* known miRNAs expression profile in female; **C** Fitting chart of three clusters miRNAs expression profile in female; **D** Heatmap of 79 *S. japonicum* known miRNAs expression profile in male; **E** Three clusters of 79 *S. japonicum* known miRNAs expression profile in male; **F** Fitting chart of three clusters miRNAs expression profile in male.Cluster 1: miRNAs had high expression from 14 to 18 dpi. Cluster 2: miRNAs had high expression from 18 to 22 dpi. Cluster 3: miRNAs had high expression from 26 to 28 dpi. MiRNAs expression profile (log scale) data was normalized reads, which were generated by the quantifier module in miRDeep2 from sequencing reads and formed by the fold change formula.

The expression profiles of 79 known miRNAs (Figure 2, Additional file 5) showed gender and stage differentiation. We validated 12 randomly selected miRNAs for expression by qRT-PCR, which showed miRNA relative expression by qRT-PCR was consistent with the profiles showed by miRNA sequencing (Additional files 6 and 7). The expression profile of all 79 known miRNAs (Figure 2) were grouped into three clusters in each gender (Table 1). In the female cluster 1 (F-cluster 1) (Figure 2B, C), the expression of 20 miRNAs elevated continuously from 14 to 18 dpi, then declined from 20 to 28 dpi. In F-cluster 2 (Figure 2B and C), 25 miRNAs maintained high expression from 18 to 22 dpi. In F-cluster 3 (Figure 2B and C), most of 34 miRNAs presented an abrupt high expression profile at 26 dpi and 28 dpi. In the male expression profile (Figure 2D) the miRNA expression was also grouped into three similar clusters. In M-cluster 1 (Figure 2E and F) 37 miRNAs were expressed at high levels from 14 to 20 dpi. In M-cluster 2 (Figure 2E and F), 22 miRNAs were expressed at high levels from 20 to 26 dpi. For M-cluster 3 (Figure 2E and F), most of those 20 miRNAs were expressed at low levels from 14 to 26 dpi while at 28 dpi their expression increased abruptly. These results provided a straightforward overview of similar miRNAs expression patterns of cluster 1, 2 and 3 in male and female. Cluster 1 was corresponding to the pairing stage (14–20 dpi), cluster 2 to the germ cell development and sexual maturation stage (20–26 dpi) and cluster 3 to egg production stage (24–28 dpi).

**Table 1:** **The 79 known** ***S. japonicum*** **miRNAs clusters in female and male**

MiRNAs cluster	Female	Male
Cluster 1	Total: 20 sja-miR-10-5p, sja-miR-124-5p, sja-miR-2162-5p, sja-miR-2c-5p, sja-miR-2c-3p, sja-miR-2d-5p, sja-miR-3479-5p, sja-miR-3482-5p, sja-miR-3484-3p, sja-miR-3494, sja-miR-7-3p, sja-miR-190-3p, sja-miR-2a-3p, sja-miR-2e-5p, sja-miR-310, sja-miR-3483-3p, sja-miR-3498, sja-miR-3501, sja-miR-3503, sja-miR-71b-3p	Total: 37 sja-miR-2b-3p, sja-miR-2c-3p, sja-miR-3485-5p, sja-miR-3485-3p, sja-miR-3489, sja-miR-3491, sja-miR-3498, sja-miR-3500, sja-miR-71b-3p, sja-miR-2162-5p, sja-miR-2d-3p, sja-miR-2e-5p, sja-miR-3483-3p, sja-miR-3486-5p, sja-miR-277, sja-miR-3490, sja-miR-3492, sja-miR-3493, sja-miR-3494, sja-miR-3497, sja-miR-3499, sja-miR-3501, sja-miR-3502, sja-miR-3504, sja-miR-3506, sja-miR-36-5p, sja-miR-71b-5p, sja-miR-7-3p, sja-miR-124-5p, sja-miR-190-3p, sja-miR-2a-3p, sja-miR-2c-5p, sja-miR-2d-5p, sja-miR-310, sja-miR-3479-5p, sja-miR-3481-5p, sja-miR-3485
Cluster 2	Total: 25 sja-let-7, sja-miR-219-5p, sja-miR-2a-5p, sja-miR-2e-3p, sja-miR-31-5p, sja-miR-31-3p, sja-miR-3507, sja-miR-36-5p, sja-miR-10-3p, sja-miR-124-3p, sja-miR-133, sja-miR-1, sja-miR-219-3p, sja-miR-2b-3p, sja-miR-2d-3p, sja-miR-307, sja-miR-3480-5p, sja-miR-3482-3p, sja-miR-3484-5p, sja-miR-36-3p, sja-miR-61, sja-miR-71a, sja-miR-71b-5p, sja-miR-8-5p, sja-miR-8-3p	Total: 22 sja-miR-124-3p, sja-miR-125a, sja-miR-190-5p, sja-miR-1, sja-miR-3488, sja-miR-3496, sja-miR-3505, sja-miR-61, sja-miR-8-3p, sja-let-7, sja-miR-10-3p, sja-miR-133, sja-miR-2162-3p, sja-miR-219-5p, sja-miR-219-3p, sja-miR-2e-3p, sja-miR-307, sja-miR-3479-3p, sja-miR-3482-3p, sja-miR-3487, sja-miR-7-5p, sja-bantam
Cluster 3	Total: 34 sja-miR-125b, sja-miR-3481-5p, sja-miR-3485-3p, sja-miR-3486-5p, sja-miR-3486-3p, sja-miR-3487, sja-miR-3488, sja-miR-3489, sja-miR-3492, sja-miR-3495, sja-miR-3499, sja-miR-3506, sja-miR-7-5p, sja-bantam, sja-miR-125a, sja-miR-190-5p, sja-miR-2162-3p, sja-miR-2b-5p, sja-miR-3479-3p, sja-miR-3480-3p, sja-miR-3481-3p, sja-miR-3483-5p, sja-miR-3485-5p, sja-miR-3490, sja-miR-3491, sja-miR-3496, sja-miR-3497, sja-miR-3500, sja-miR-3502, sja-miR-3504, sja-miR-277, sja-miR-3505, sja-miR-8185, sja-miR-3493	Total: 20 sja-miR-10-5p, sja-miR-2a-5p, sja-miR-2b-5p, sja-miR-31-3p, sja-miR-3480-5p, sja-miR-3482-5p, sja-miR-3483-5p, sja-miR-3484-3p, sja-miR-71a, sja-miR-8185, sja-miR-125b, sja-miR-3481-3p, sja-miR-3503, sja-miR-36-3p, sja-miR-8-5p, sja-miR-3480-3p, sja-miR-3507, sja-miR-31-5p, sja-miR-3484-5p, sja-miR-3486-3p

### Functional enrichment of predicted miRNA targets

**Figure 3:**
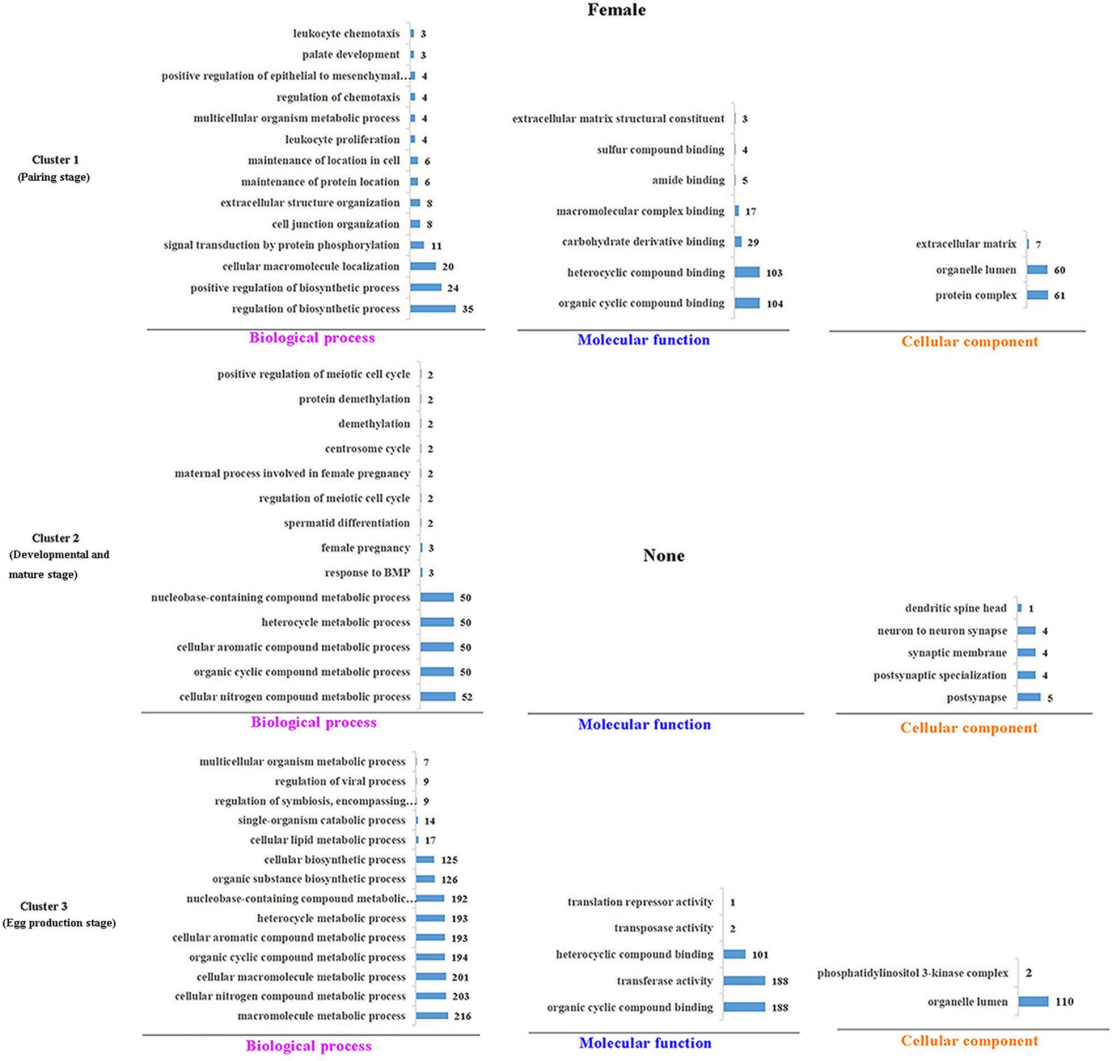
**GO enrichments of miRNA target genes of clusters in female.** Each cluster GO terms were processed with Blast2GO (v4.2) GO terms were divided into three categories: biological process, molecular function, cellular component. The number of targets in each cluster category was shown in bar charts.

**Figure 4:**
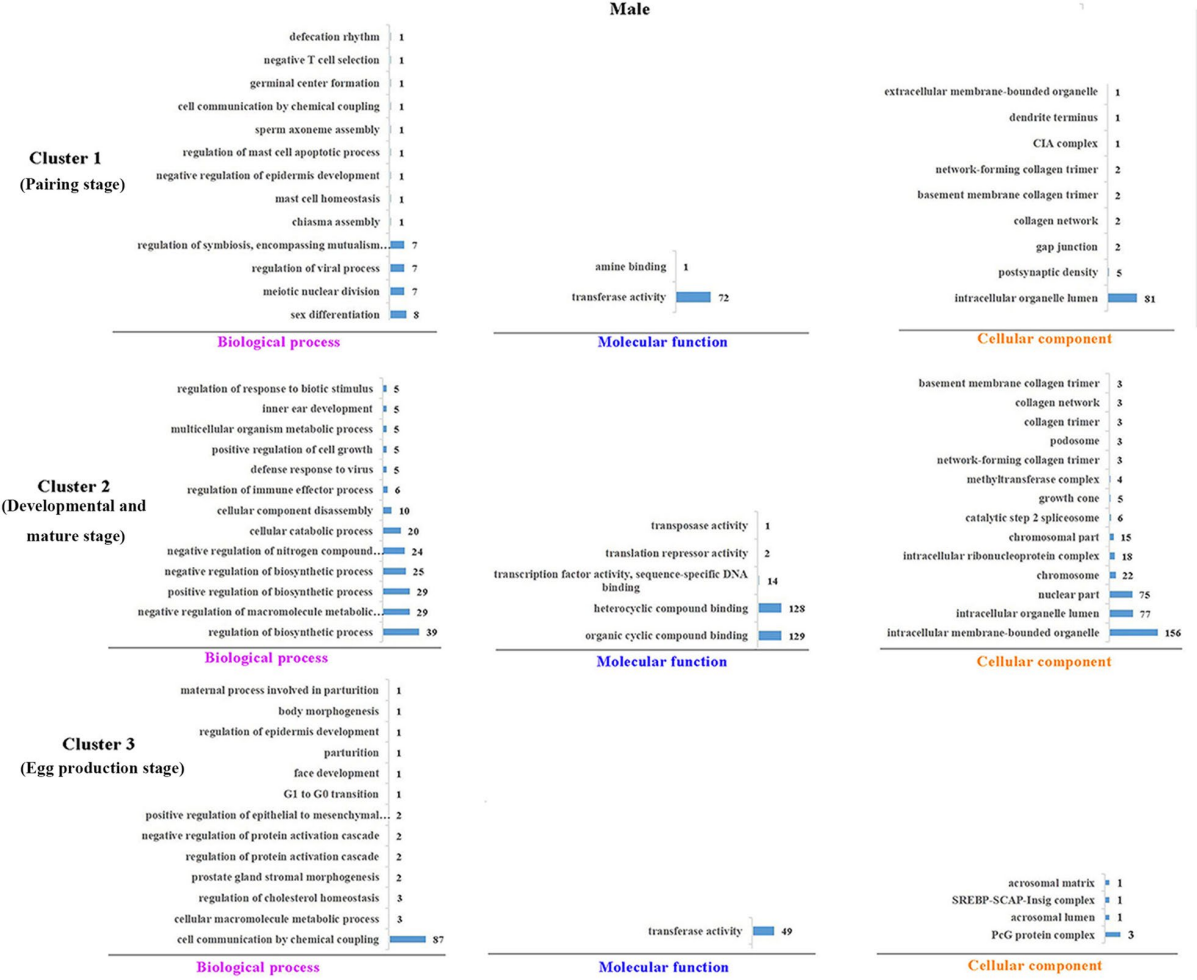
**GO enrichments of miRNA target genes of clusters in male.** Each cluster GO terms were processed with Blast2GO v4.2. GO terms were divided into three categories: biological process, molecular function, cellular component. The number targets in each cluster category were shown in bar charts.

Overlap targets were predicted in RNAhybrid and miRanda considered as the potential targets of miRNA (Additional file 8), we selected 3 miRNAs (kept high expression level during 18 to 22 dpi) which had the potential regulation ability to the reproductive development. For identifying target genes of 3 miRNAs, we selected the genes containing the combine sites with miRNA in the 3′UTR, it is common view of miRNA potential target combine sites. The validation were performed in dual luciferase reporter system (Additional file 9). Then, miRNA targets were used to process the functional enrichments. The major enrichments showed in Figure 3 (female) and Figure 4 (male). In female, as to biological process assignment: F-cluster 1 was mainly enriched in regulation of biosynthetic processes, cellular macromolecule localization, and signal transduction by protein phosphorylation (Figure 3). F-cluster 2 was mainly enriched in metabolic processes. F-cluster 3 was mainly enriched in metabolic process and biosynthetic process (Figure 3). In males, as to the biological process assignment: In male, M-cluster 1 was mainly enriched in sex differentiation, meiotic nuclear division, regulation of viral process, regulation of symbiosis (encompassing mutualism through parasitism) (Figure 4). M-cluster 2 was mainly enriched in regulation of the biosynthetic, metabolic and catabolic processes. F-cluster 3 was mainly enriched in cellular macromolecule metabolic processes (Figure 4). As to the molecular function assignment, transferase activity was an arresting enrichment, which were enriched in F-cluster 3 (188 targets), M-cluster 1 (79 targets) and M-cluster 3 (49 targets).

**Figure 5:**
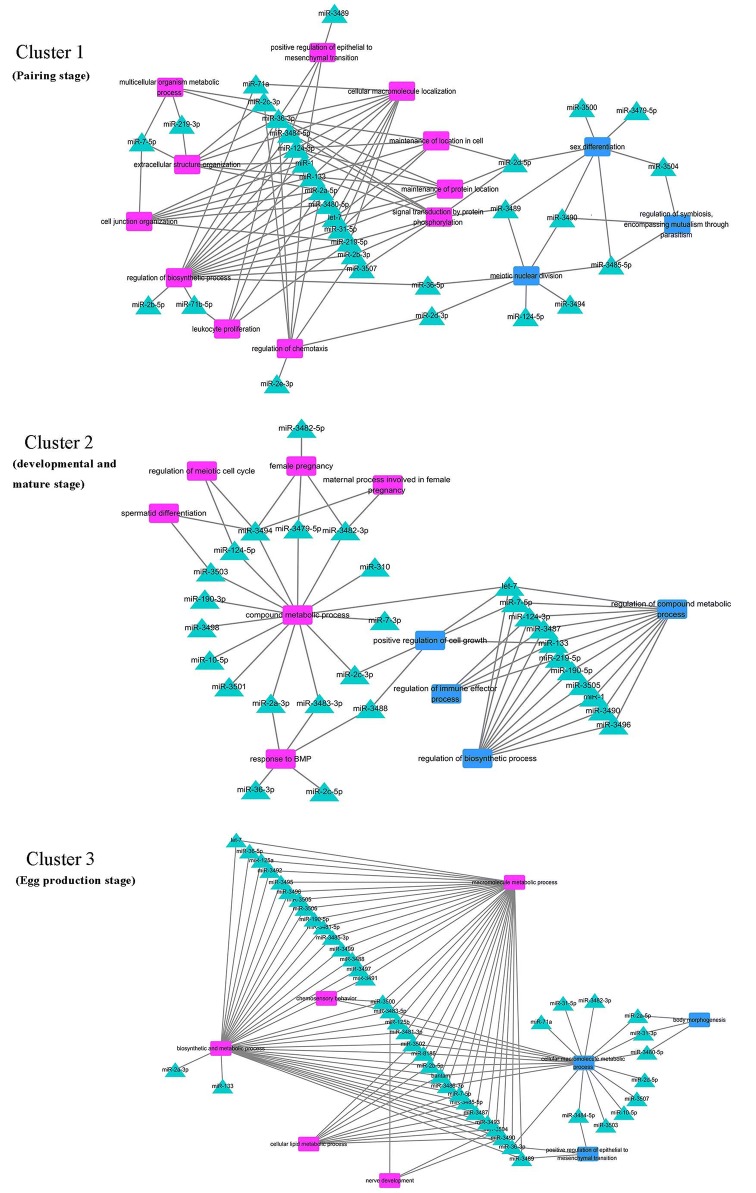
**Network of cluster miRNA-targeted genes GO terms in female and male.** The relationship between miRNAs and their targets’ GO enrichment in each cluster were processed in cytoscape3.6.0. Purple, square represented enrichment in female. Blue, square represented enrichment in male. Turquoise, triangle represented miRNA. Cluster 1: pairing stage; cluster 2: developmental and sexual maturation stage; cluster 3: egg production stage.

Except for the major enrichment, there were also some specific enrichments in female and male, which were interesting and worthy of being concerned. Detailed information was showed in Table 2. In a summary, enrichments of each cluster in female and male were different. We discriminated and concluded the enrichments in female and male, respectively. Then we listed which miRNAs regulated those enrichments and showed their network in Figure 5 and Additional file 10.

**Table 2:** **Difference of miRNAs cluster targets enrichment between female and male**

Gender	Stages	Specific functional enrichments
Female	Cluster 1 (pairing stage)	Regulation of chemotaxis Leukocyte proliferation Cell junction organization Regulation of epithelial to mesenchymal transition
	Cluster 2 (developmental and mature stage)	Response to BMP (bone morphogenetic protein) Female pregnancy Spermatid differentiation Regulation of meiotic cell cycle
	Cluster 3 (egg production stage)	Nerve development Chemosensory behavior
Male	Cluster 1 (pairing stage)	Sex differentiation Meiotic nuclear division
	Cluster 2 (developmental and mature stage)	Regulation of immune effector process Regulation of cell growth Regulation of response to biotic stimulus
	Cluster 3 (egg production stage)	Cell communication by chemical coupling Regulation of cholesterol homeostasis Regulation of protein activation cascade Body morphogenesis

### Network analysis between miRNAs and their targets enrichment

In order to further understand the multiple regulation roles of miRNAs during different developmental stages, we analyzed the network between miRNAs and their target enrichments in cluster 1, cluster 2 and cluster 3 (Figure 5, Additional file 10), respectively. The network showed 31 miRNAs participated in the regulation in cluster 1 (pairing stage). Among them, 20 miRNAs were specifically enriched in females, 7 miRNAs were specifically enriched in males, and 4 miRNAs were enriched both in females and males. Notably in females, 5 miRNAs regulated the leukocyte proliferation, 7 miRNAs regulated the chemotaxis, and 9 miRNAs regulated the signal transduction by protein phosphorylation. These 15 miRNAs (Figure 5, Additional file 10) might play a key role in the stimulation or attraction of female-male pairing, communication and immune response in parasite–host during pairing stage. In males, 7 miRNAs regulated sex differentiation, and 7 miRNAs regulated meiotic nuclear division. It was suggested that miRNAs may be involved in male sexual development regulation at the early paring stage [44]. In cluster 2 (developmental and mature stage), 29 miRNAs participated the developmental and sexual maturation stage. 15 miRNAs were only enriched in females, 10 miRNAs were only enriched in males, and 4 miRNAs were enriched both in females and males. Notably, 6 miRNAs regulated meiotic cell cycle, pregnancy process in females. 5 miRNAs regulated the cell growth in males. In cluster 3 (egg production stage), 45 miRNAs participated in regulation during the egg production stage, the items of enrichments were less in female and male comparing with cluster 1 and 2. However, 34 miRNAs in female and 21 miRNAs in male were enriched in metabolic processes, which was the largest number of miRNAs observed in a process (Figure 5, Additional file 10). These miRNAs might regulate numerous material synthesis and metabolism for egg production. Notably, 2 miRNAs were enriched in chemosensory behavior, 3 miRNAs were enriched in nerve development, 3 miRNAs were enriched in body morphogenesis and 3 miRNAs were enriched in regulation of epithelial to mesenchymal transition. These 7 miRNAs in cluster 3 might play specific role during interplay in female-male and female specialized function of egg production (Figure 5, Additional file 10). Above information may provide many new clues to investigating the regulation mechanism of male and female sexual development, which deserves further attention and experimental validation.

## Discussion

This study focused on *S. japonicum* 79 known miRNAs profile (14 to 28 dpi), and the results suggested that *S. japonicum* miRNAs had three similar expression clusters in male and female from pairing, development, sexual maturation to egg production. The stage related miRNA clusters may regulate specific functions and play distinct roles in order to accomplish *S. japonicum* parasitism in host during pairing, sex-maturation and production. MiRNA is a potential factor in male–female and parasite–host interplay. Our study provided a comprehensive miRNA analysis with the goal of understanding the molecular role for miRNAs during developmental events of the schistosome life cycle.

We also looked at the target functional enrichments of “the spermatid differentiation” in females, which was interesting. It has previously been reported [44] that cells in the male seminal vesicle were identified as early stages of spermatogenesis, which were immature sperm, while the mature sperms were found in female seminal vesicle and oviduct. This phenomenon indicated that the spermatid maturation process might occur in female. Another possibility of this phenomenon was the sperm maturation occurred in male and the mature sperms were stored in the end of excretory bladder and then given to the female. We couldn’t observed the whole process and couldn’t improve this suppose.

Transferase activity are involved in hundreds of different biochemical pathways throughout the whole biological process, and are integral to some of life’s most important processes. Our results in transferase activity enrichments in F-cluster 3 (188, 39%), M-cluster 1 (72, 99%), M-cluster 3 (49, 100%). showed different transferase activity groups enriched in female and male. Results (Additional files 11, 12) showed 53.1% (26 of 49) of the targets were specific in M-cluster 3, 18.1% (34 of 188) targets were specific in F-cluster 3, 33.3% targets (24 of 72) were specific in M-cluster 1. Next, we also analyzed which miRNAs regulated those transferase group, results (Additional file 13) showed 10 miRNAs regulated transferase activity were specific in M-cluster 3, 7 miRNAs were specific in F-cluster 3, 12 miRNAs were specific in M-cluster 1. There were also different subcategories (Additional file 14) of transferase were enriched in F-cluster 3, M-cluster 1 and M-cluster 3. The rich transferase family member of the targets suggested that miRNA can regulate many important biological processes through post-translational modification [45–48] which included of methyltransferase, acetyltransferase, glucosyltransferase, galactosyltransferase, phosphoribosyl transferase, aminotransferase, mannosyl transferase, ribosyl transferase, cytidylyl transferase, pyrophospho kinase, et al. Especially, methyltransferase and acetyltransferase were key regulatory factors in epigenetics via DNA methylation, histone methylation and histone acetylation. Okada et al. [49] reported that histone demethylase JHDM2A is critical for *Tnp1* and *Prm1* transcription and spermatogenesis, O’Carroll et al. [50] reported that histone H3 methyltransferase gene-*Suv39h2* that displays testis-specific expression, Tachibana et al. [51] reported G9a histone methyltransferase plays a dominant role in euchromatic histone H3 lysine 9 methylation and is essential for early embryogenesis. Disorder in histone methylation or acetylation during spermatogenesis directly affected the establishment and maintenance of epigenetic modifications, leading to spermatogenic cell abnormalities and even infertility. Even though those reports were all carried out in mammalian systems, our results indicated methyltransferase activity regulated by miRNA in schistosome might play important developmental role via post-translational modification, e.g. methylation and acetylation.

## Additional files

**Additional file 1.** **Flow charts of data analysis.**

**Additional file 2.** **Classification and reads in** ***S. japonicum*** **small RNAs sequencing.**

**Additional file 3.** **Percentage of small RNAs mapped to database and length of small RNAs in** ***S. japomicum*** **miRNA sequencing.** A: the percentage of sequences in small RNA sequencing mapped to the *S. japonicum* genome (left) and miRNA database (right). B: the length of small RNAs in female and male.

**Additional file 4.** **Candidate miRNAs mature sequences, star sequences, precursor sequences.**

**Additional file 5.** **Fold change of known miRNAs in 8 developmental points, totally 48 samples.**

**Additional file 6.** **Validation of miRNAs expression in miRNAs sequencing by qRT-PCR.** A: the expression of 12 random miRNAs in sequencing normalized data. B: the relative expression of random 12 miRNAs by qRT-PCR.

**Additional file 7.** **Primers used in miRNA qRT-PCR.**

**Additional file 8.** **The number of potential targets of 79 known miRNAs predicted in RNAhybrid and miRanda.**

**Additional file 9.** **Validation miRNA target’s validity using dual luciferase report system.** 6 targets (combine sites in the targets 3’ UTR) of 3 miRNAs (sja-miR-125a, sja-miR-125b and sja-miR3487, high expression during 18–22 dpi) were selected to validate the targets validity using pmirGLO luciferase report system. Results showed 4/6 (66.7%) predicted targets were successfully validated. Mimics represented miRNA mimics; NC represented NC mimics; blank represented no chemically synthetic oligonucleotides. Target vector represented including miRNA binding site fragment recombinant plasmid; WT vector represented wild type vector (pmirGLO vector). NC mimics and WT vector were set as negative control. Data were presented as the mean ± SD of triplicate independent experiments. “*”: *p* < 0.05; “**”: *p* < 0.001 (Student’s t*-*test), NS: not significant.

**Additional file 10.** **miRNAs and their targets enrichments in different developmental stages in male and female.**

**Additional file 11.** **Network of transferase activity and their targets in female and male.** The relationship between transferase activity and their targets in female and male were processed in cytoscape3.6.0. Purple, square represented enrichments in female. Blue, square represented enrichments in male. Turquoise, oval represented miRNA targets.

**Additional file 12.** **Network of transferase activity and their miRNAs in female and male.** The relationship between transferase activity and targets in female and male were processed in cytoscape3.6.0. Purple, square represented enrichments in female. Blue, square represented enrichments in male. Turquoise, triangle represented miRNA.

**Additional file 13.** **Transferase activity enriched in female and male cluster.**

**Additional file 14.** **MiRNAs regulated different transferase activities in female and male.**
