# Supplementary figures and images for: Comprehensive analysis of miRNA profiles reveals the role of Schistosoma japonicum miRNAs at different developmental stages
Source: Vet Res. 2019 Apr 4;50:23. doi: 10.1186/s13567-019-0642-2 (PMC6449929; doi:10.1186/s13567-019-0642-2)

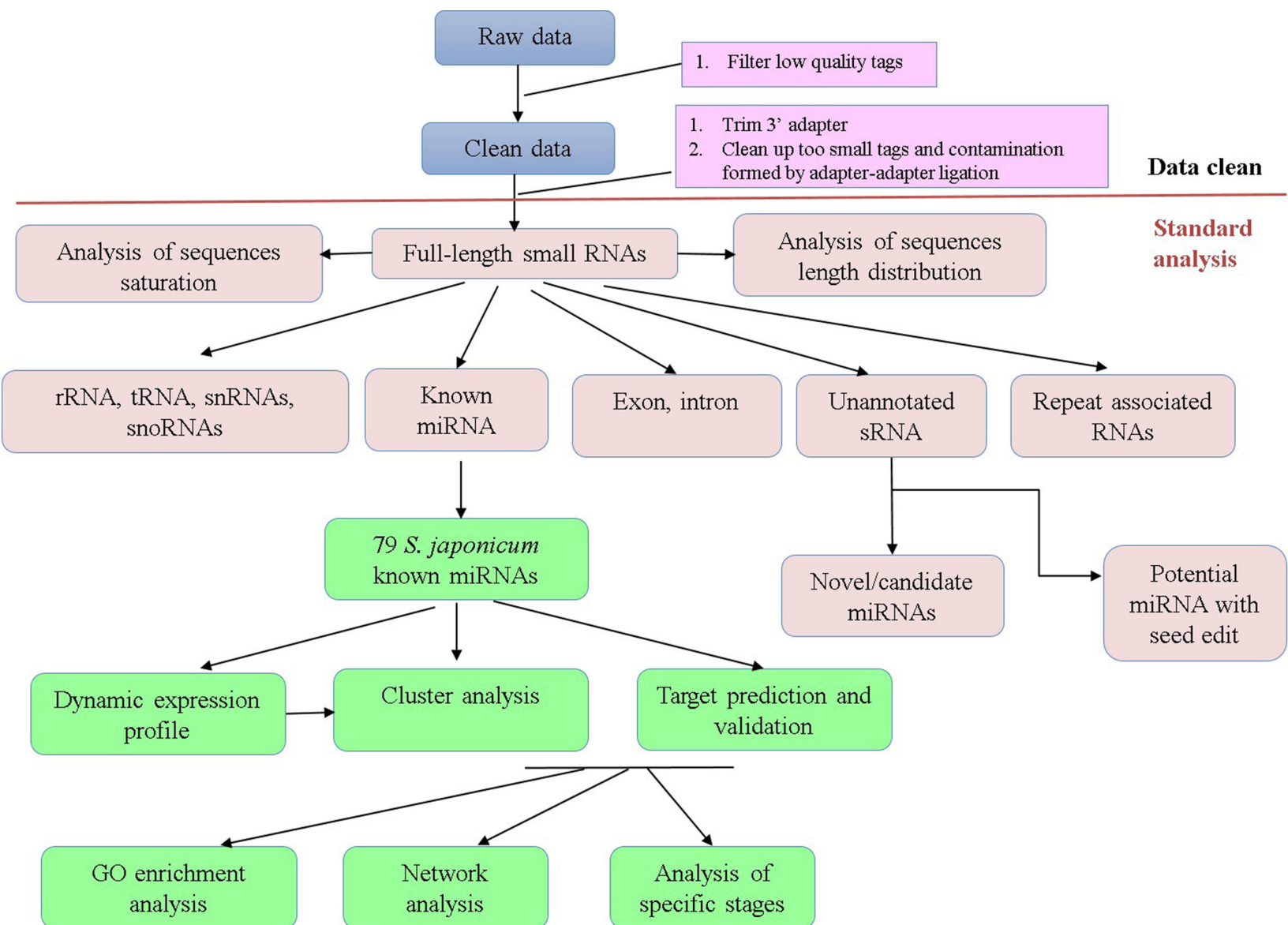

Supplement: Supplementary file 1 — Additional file 1. Flow charts of data analysis. [file 13567_2019_642_MOESM1_ESM.pdf]

A

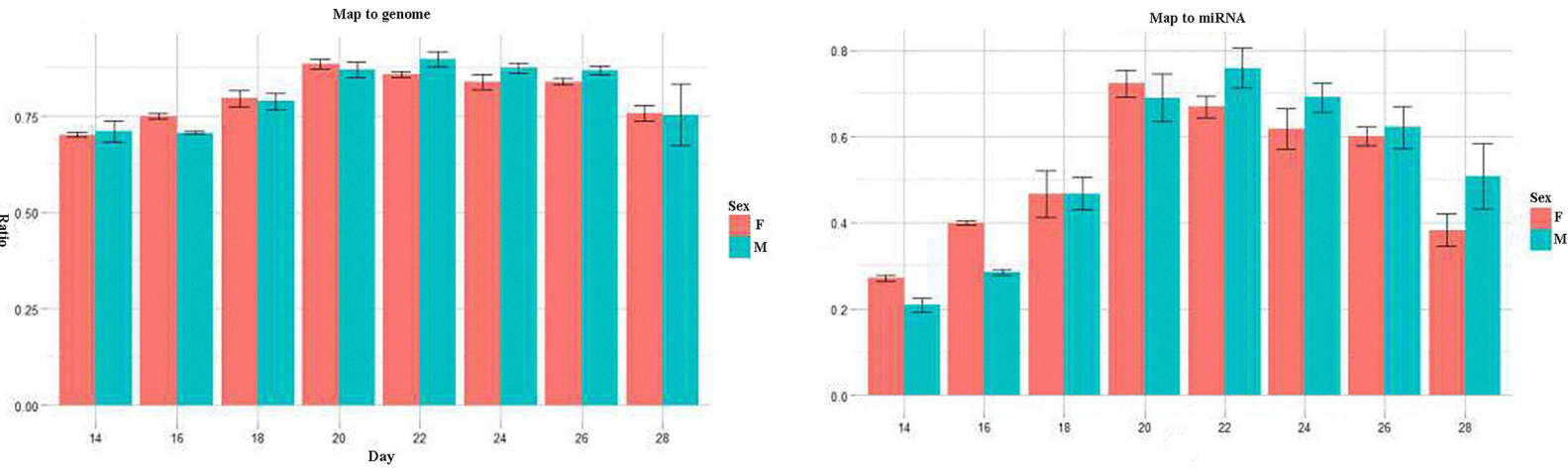

B

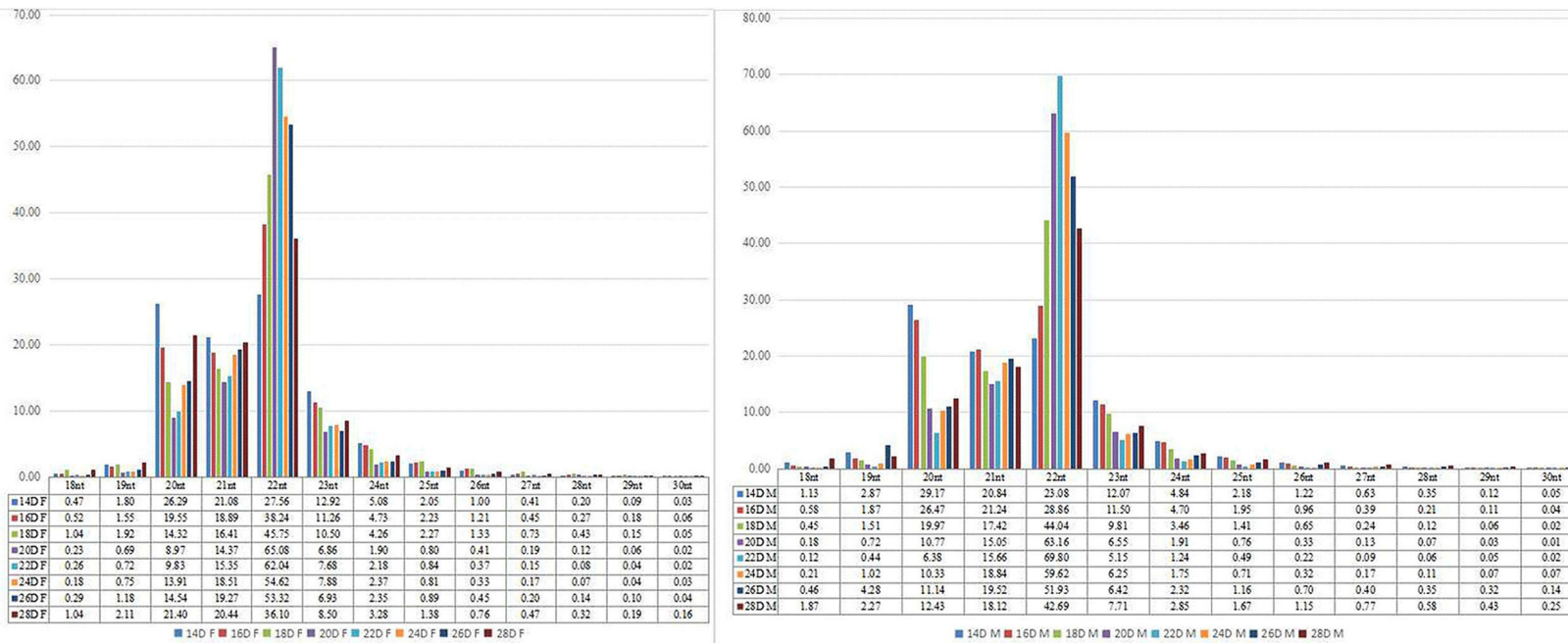

Supplement: Supplementary file 3 — Additional file 3. Percentage of small RNAs mapped to database and length of small RNAs in S. japomicum miRNA sequencing. A: the percentage of sequences in small RNA sequencing mapped to the S. japonicum genome (left) and miRNA database (right). B: the length of small RNAs in female and male. [file 13567_2019_642_MOESM3_ESM.pdf]

A. miRNA sequencing

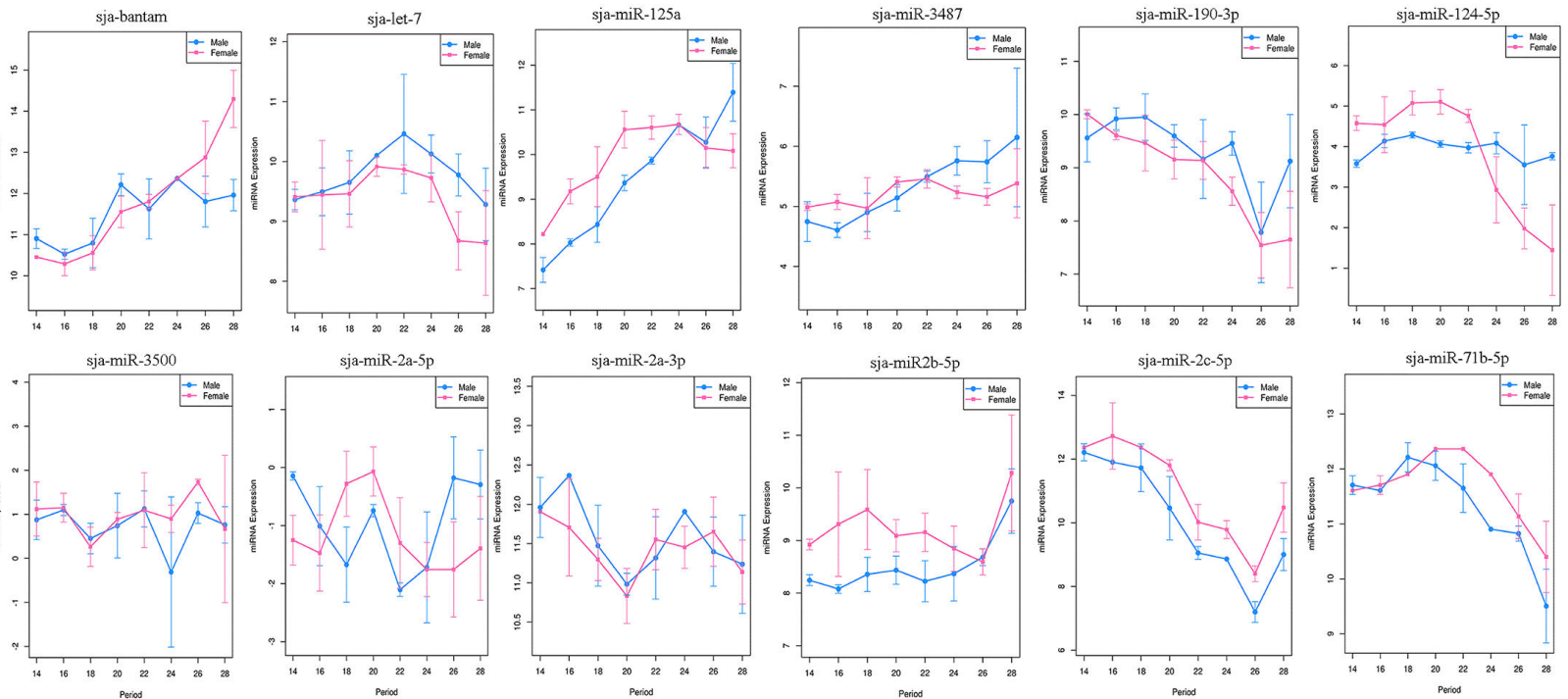

B. qRT-PCR

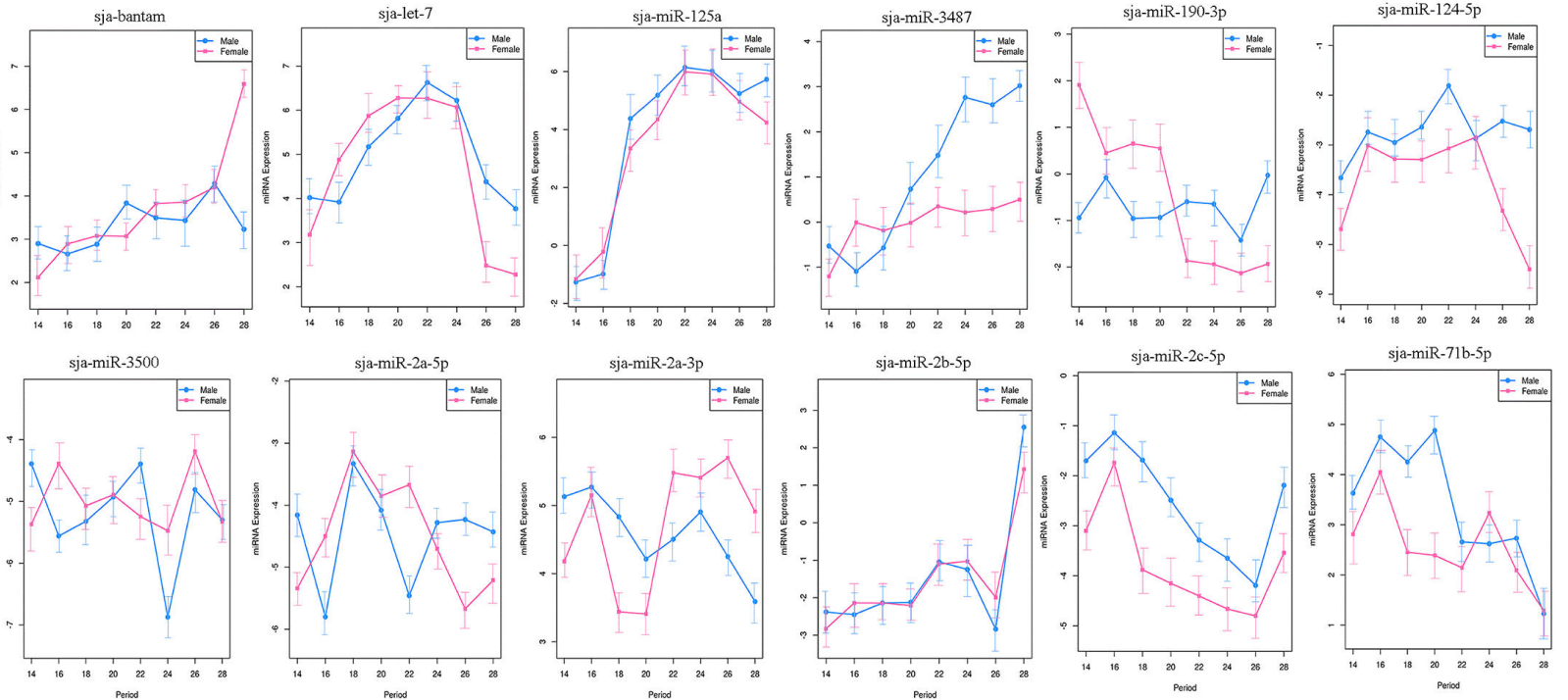

Supplement: Supplementary file 6 — Additional file 6. Validation of miRNAs expression in miRNAs sequencing by qRT-PCR. A: the expression of 12 random miRNAs in sequencing normalized data. B: the relative expression of random 12 miRNAs by qRT-PCR. [file 13567_2019_642_MOESM6_ESM.pdf]

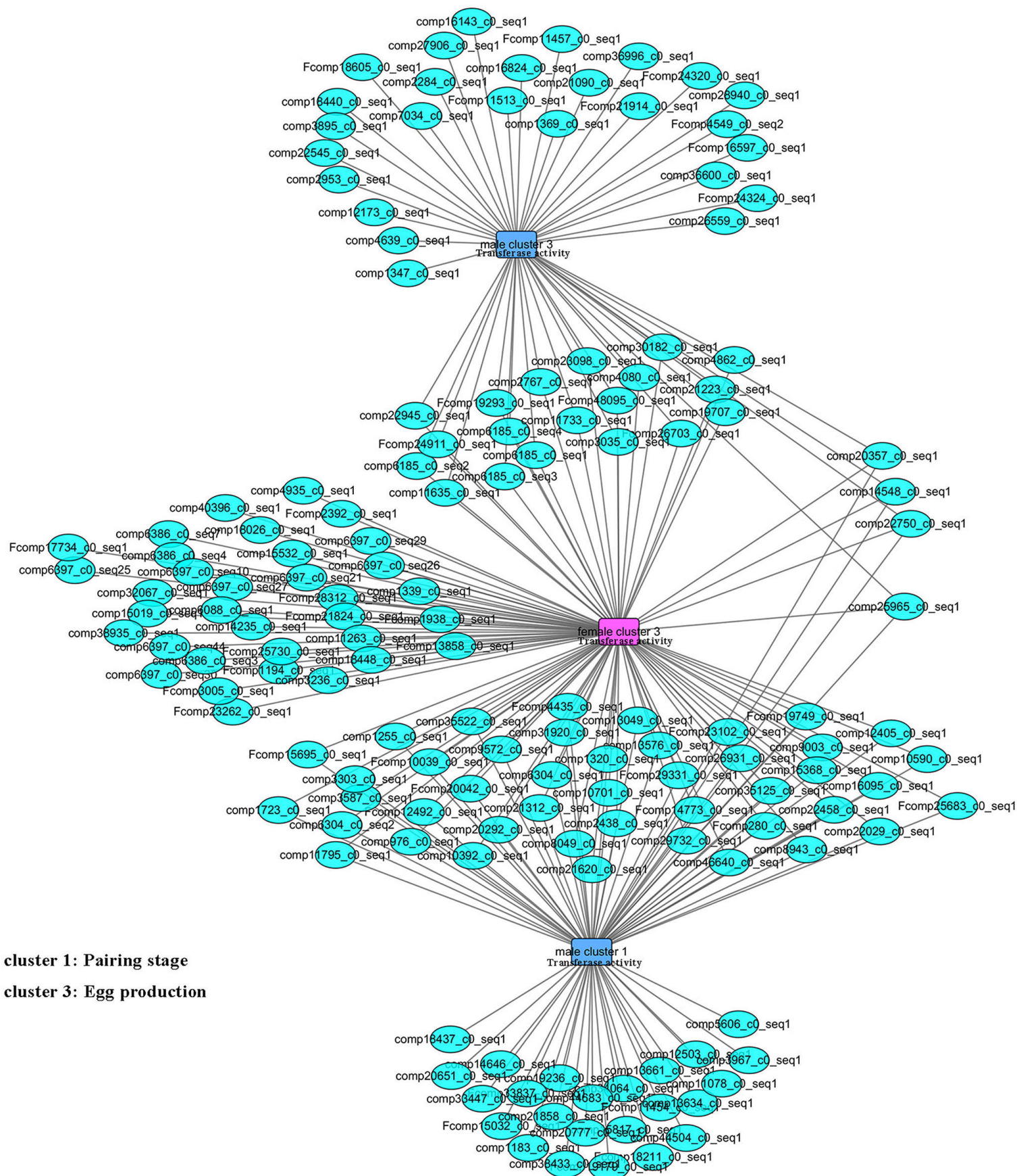

cluster 1: Pairing stage  
cluster 3: Egg production

Supplement: Supplementary file 11 — Additional file 11. Network of transferase activity and their targets in female and male. The relationship between transferase activity and their targets in female and male were processed in cytoscape3.6.0. Purple, square represented enrichments in female. Blue, square represented enrichments in male. Turquoise, oval represented miRNA targets. [file 13567_2019_642_MOESM11_ESM.pdf]

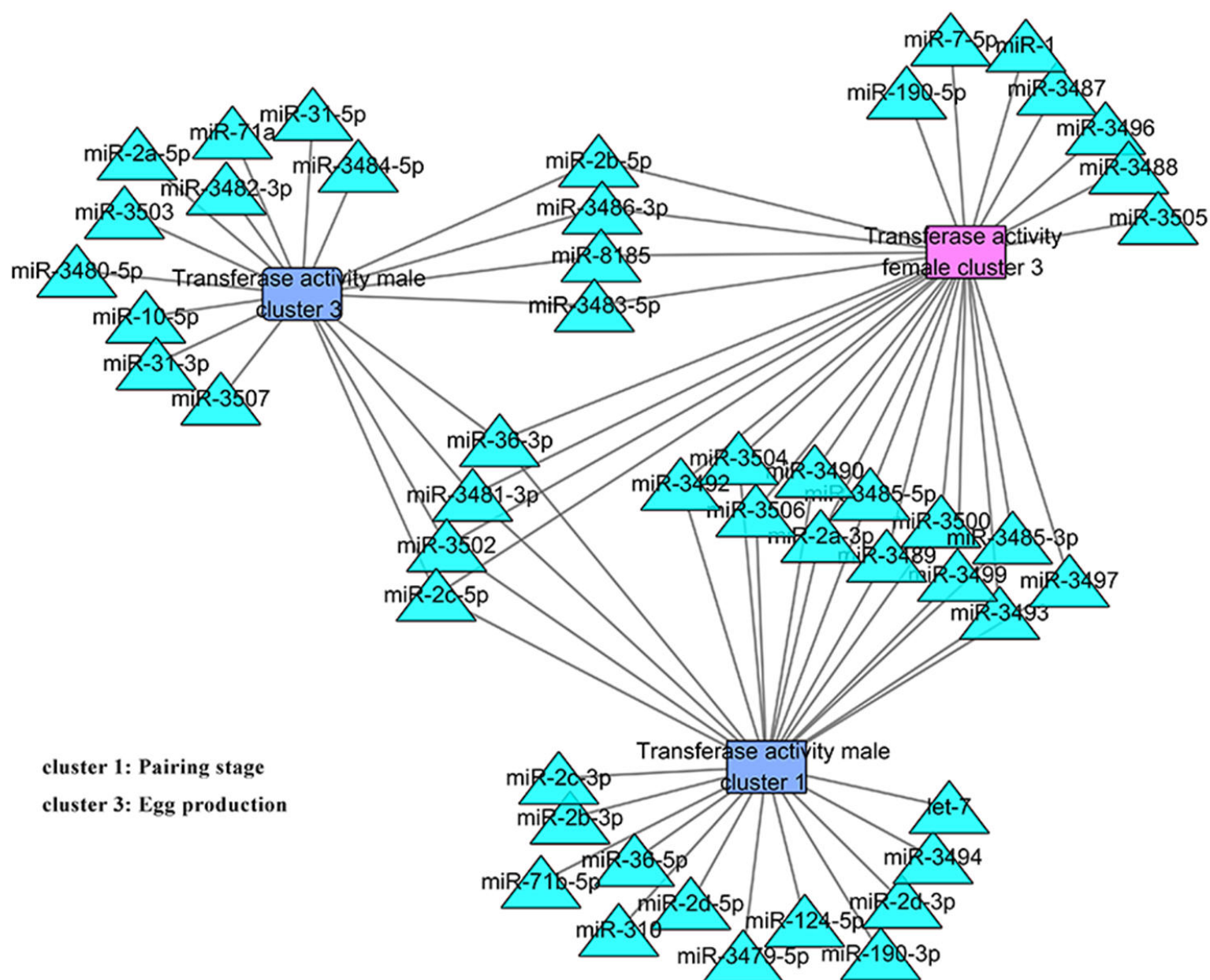

Supplement: Supplementary file 12 — Additional file 12. Network of transferase activity and their miRNAs in female and male. The relationship between transferase activity and targets in female and male were processed in cytoscape3.6.0. Purple, square represented enrichments in female. Blue, square represented enrichments in male. Turquoise, triangle represented miRNA. [file 13567_2019_642_MOESM12_ESM.pdf]
