# Supplementary material for: Comprehensive analysis of miRNA profiles reveals the role of Schistosoma japonicum miRNAs at different developmental stages
Source: Vet Res. 2019 Apr 4;50:23. doi: 10.1186/s13567-019-0642-2 (PMC6449929; doi:10.1186/s13567-019-0642-2)
Supplement: Supplementary file 2 — Additional file 2. Classification and reads in S. japonicum small RNAs sequencing. [file 13567_2019_642_MOESM2_ESM.doc]

**Additional file 2: Classification and reads in *S. japonicum* small RNAs sequencing.**

| **gender** | **categories** | **14 dpi** | **16 dpi** | **18 dpi** | **20 dpi** | **22 dpi** | **24 dpi** | **26 dpi** | **28 dpi** |
| --- | --- | --- | --- | --- | --- | --- | --- | --- | --- |
| Female | Exon(antisense) | 357092 | 251110 | 118527 | 107815 | 86638 | 156999 | 136231 | 284633 |
|  | Exon(sense) | 417787 | 341200 | 324062 | 165570 | 103169 | 211591 | 210234 | 478988 |
|  | Intron(antisense) | 559974 | 460376 | 209109 | 179142 | 137421 | 262865 | 237198 | 575156 |
|  | Intron(sense) | 1059441 | 891946 | 663143 | 327908 | 223489 | 425803 | 456370 | 1003491 |
|  | miRNA | 8024446 | 11743579 | 9059411 | 19248296 | 12532654 | 16897780 | 16116902 | 12197289 |
|  | rRNA | 454410 | 573208 | 703840 | 208587 | 77246 | 121444 | 206950 | 630786 |
|  | repeat | 6350150 | 4464937 | 2131661 | 1574025 | 1250263 | 2589632 | 2744688 | 5535202 |
|  | snRNA | 28837 | 35341 | 31357 | 9732 | 4140 | 5398 | 5839 | 27792 |
|  | snoRNA | 2111 | 1566 | 825 | 613 | 395 | 1075 | 1134 | 1608 |
|  | tRNA | 111156 | 127719 | 85974 | 52120 | 20913 | 68423 | 165571 | 286893 |
|  | unannotation | 12345616 | 10095210 | 5317506 | 4438562 | 3572582 | 6007223 | 5640082 | 11380483 |
|  | Total | 29711020 | 28986192 | 18645415 | 26312370 | 18008910 | 26748233 | 25921199 | 32402321 |
| Male | Exon(antisense) | 384121 | 377632 | 189668 | 147945 | 40657 | 131088 | 114149 | 142862 |
|  | Exon(sense) | 562133 | 412904 | 215839 | 168589 | 53735 | 180581 | 259566 | 336029 |
|  | Intron(antisense) | 568474 | 563678 | 273840 | 216067 | 64626 | 195841 | 198743 | 260818 |
|  | Intron(sense) | 1414260 | 1018719 | 469247 | 339798 | 105096 | 364738 | 488295 | 667169 |
|  | miRNA | 6173140 | 8769482 | 8566803 | 19521356 | 12024871 | 18391386 | 16001535 | 13782722 |
|  | rRNA | 1159093 | 341475 | 145701 | 60320 | 32186 | 155889 | 469931 | 1041006 |
|  | repeat | 6985139 | 6424588 | 2880437 | 1992577 | 565157 | 1793366 | 1871259 | 2240278 |
|  | snRNA | 40353 | 20352 | 8469 | 3680 | 1090 | 6131 | 10929 | 36906 |
|  | snoRNA | 2688 | 3463 | 1005 | 606 | 408 | 604 | 554 | 2483 |
|  | tRNA | 145993 | 106699 | 62920 | 39439 | 17878 | 155102 | 1136448 | 275372 |
|  | unannotation | 12432663 | 12595707 | 6290561 | 5199745 | 1707043 | 4722051 | 5117171 | 8364783 |
|  | Total | 29868057 | 30634699 | 19104490 | 27690122 | 14612747 | 26096777 | 25668580 | 27150428 |
