# Supplementary material for: Comprehensive analysis of miRNA profiles reveals the role of Schistosoma japonicum miRNAs at different developmental stages
Source: Vet Res. 2019 Apr 4;50:23. doi: 10.1186/s13567-019-0642-2 (PMC6449929; doi:10.1186/s13567-019-0642-2)
Supplement: Supplementary file 7 — Additional file 7. Primers used in miRNA qRT-PCR. [file 13567_2019_642_MOESM7_ESM.doc]

**Additional file 7: Primers used in qRT-PCR**

| **miRNA name** | **primers** |
| --- | --- |
| sja-bantam | TGAGATCGCGATTAAAGCTGGT |
| sja-let-7 | GGAGGTAGTTCGTTGTGTGGT |
| sja-miR-125a | TCCCTGAGACCCTTTGATTGTC |
| sja-miR-3487 | TCCTCGAACTGTTGTGGCCA |
| sja-miR-190-3p | CAGTGACCAGACATATCCCT |
| sja-miR-124-5p | CCATTTTCCGCGATTGCCTTGATTT |
| sja-miR-3500 | AGGAGATCGGTGGTAGATTGT |
| sja-miR-2a-5p | CAGTCAATATTGGCTGATGGCA |
| sja-miR-2a-3p | TCACAGCCAGTATTGATGAACG |
| sja-miR-2b-5p | CGTCTCAAAGGACTGTGAGCCA |
| sja-miR-2c-5p | ACCCTTGTTCGACTGTGATGTG |
| sja-miR-71b-5p | TGAAAGACTTGAGTAGTGAGACG |
