# Supplementary material for: Comprehensive analysis of miRNA profiles reveals the role of Schistosoma japonicum miRNAs at different developmental stages
Source: Vet Res. 2019 Apr 4;50:23. doi: 10.1186/s13567-019-0642-2 (PMC6449929; doi:10.1186/s13567-019-0642-2)
Supplement: Supplementary file 9 — Additional file 9. Validation miRNA target’s validity using dual luciferase report system. 6 targets (combine sites in the targets 3’ UTR) of 3 miRNAs (sja-miR-125a, sja-miR-125b and sja-miR3487, high expression during 18–22 dpi) were selected to validate the targets validity using pmirGLO luciferase report system. Results showed 4/6 (66.7%) predicted targets were successfully validated. Mimics represented miRNA mimics; NC represented NC mimics; blank represented no chemically synthetic oligonucleotides. Target vector represented including miRNA binding site fragment recombinant plasmid; WT vector represented wild type vector (pmirGLO vector). NC mimics and WT vector were set as negative control. Data were presented as the mean ± SD of triplicate independent experiments. “*”: p < 0.05; “**”: p < 0.001 (Student’s t-test), NS: not significant. [file 13567_2019_642_MOESM9_ESM.pdf]

Additional file 9: predicted targets validated in dual luciferase report system.

miRNAs	targets	Binding site
Sja-miR-125a	Target 1:<br>comp4643_c0_seq1<br>description: Transmembrane and ubiquitin-like domain-containing protein 2<br>GenBank: FN319357.1	mfe: -26.3 kcal/mol; p-value: 0.073251; position 465<br>target 5' G UC A 3'<br>GCAAUCAG GGG CUCAG<br>UGUUAGUU CCC GAGUC<br>miRNA 3' U A CCU 5'
	Target 2:<br>comp6072_c0_seq2<br>description: Heterogeneous nuclear ribonucleoprotein A1<br>GenBank: AY223154.2	mfe: -25.9 kcal/mol; p-value: 0.092714; position 650<br>target 5' C UUU U UUCCAGAUAC 3'<br>ACAAUU GAG GGUUUCGGGGA<br>UGUUAG UUC CCAGAGUCCCU<br>miRNA 3' U 5'
Sja-miR-125b	Target 3:<br>comp1432_c0_seq1<br>description: hypothetical protein<br>GenBank: FN317052.1	mfe: -30.5 kcal/mol; p-value: 0.009461; position 925<br>target 5' C U 3'<br>UAUCAGUCUCAGGG<br>AUAGUCAGAGUCCC<br>miRNA 3' C GUUA U 5'
	Target 4:<br>comp8492_c0_seq1<br>description: Nucleolar protein 10<br>GenBank: FN319448.1	mfe: -27.4 kcal/mol; p-value: 0.009595; position 353<br>target 5' A C A U 3'<br>GCAA UAUCAG UUUAGGGA<br>CGUU AUAGUC GAGUCCCU<br>miRNA 3' A A 5'
Sja-miR-3487	Target 5:<br>comp6072_c0_seq1<br>description: Heterogeneous nuclear ribonucleoprotein A1<br>GenBank: AY223154.2	mfe: -29.5 kcal/mol; p-value: 0.044383; position 1001<br>target 5' A U GUCU C 3'<br>UGGCC CAACAGUUUG AGG<br>ACCGG GUUGUCAAGC UCC<br>miRNA 3' U U 5'
	Target 6:<br>comp1646_c0_seq1<br>description: deoxyribonuclease<br>GenBank: FN320927.1	mfe: -27.9 kcal/mol; p-value: 0.049085; position 668<br>target 5' C A 3'<br>GCUGCGGCGGUUCGA<br>CGGUGUUGUCAAGCU<br>miRNA 3' AC CCU 5'

6 targets of 3 miRNAs (sja-miR-125a, sja-miR-125b and sja-miR-3487) have the combine sites with miRNA in the 3’UTR of target genes, it is common view of miRNA potential target combine sites.

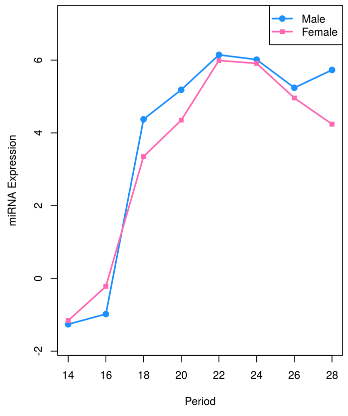

sja-miR-125a

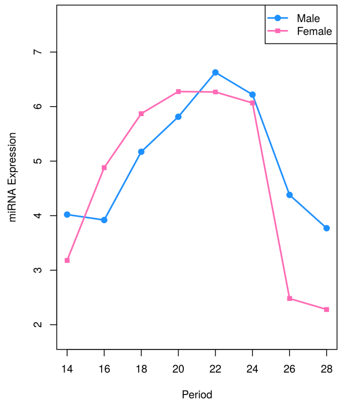

sja-miR-125b

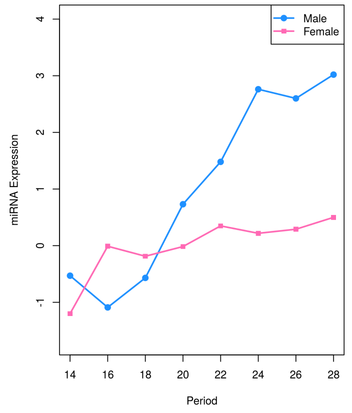

sja-miR-3487

3 miRNA (sja-miR-125a, sja-miR-125b and sja-miR-3487) all kept high expression level during 18 dpi to 22 dpi with the potential regulation ability to the reproductive development.

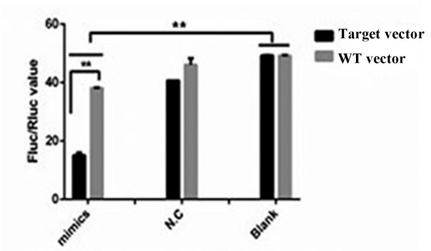

sja-miR-125a mimic + pmirGLO-target 1

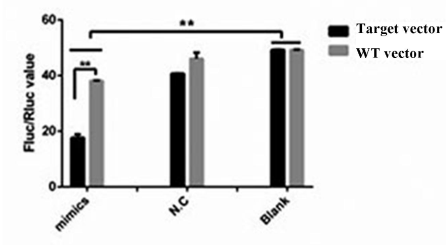

sja-miR-125a mimic + pmirGLO-target 2

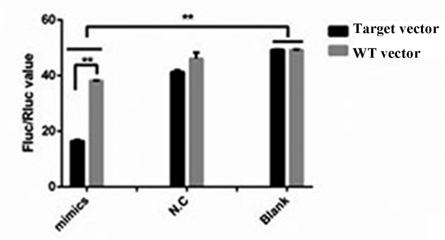

sja-miR-125b mimic + pmirGLO-target 3

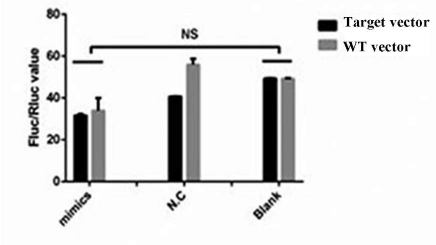

sja-miR-125b mimic + pmirGLO-target 4

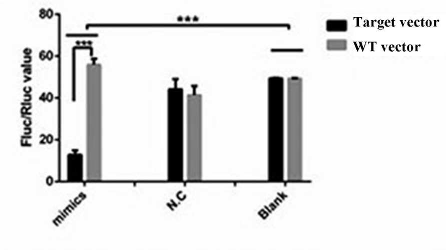

sja-miR-3487 mimic + pmirGLO-target 5

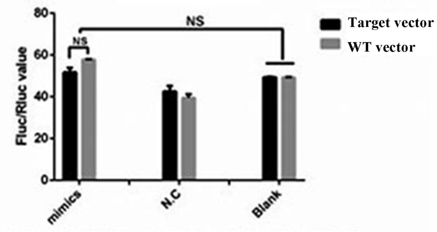

sja-miR-3487 mimic + pmirGLO-target 6

4 of 6 targets were successfully validated using dual luciferase reporter system, which illustrated that 66.67% (4/6) reliability of target prediction.
