# Supplementary material for: Comprehensive analysis of miRNA profiles reveals the role of Schistosoma japonicum miRNAs at different developmental stages
Source: Vet Res. 2019 Apr 4;50:23. doi: 10.1186/s13567-019-0642-2 (PMC6449929; doi:10.1186/s13567-019-0642-2)
Supplement: Supplementary file 10 — Additional file 10. miRNAs and their targets enrichments in different developmental stages in male and female. [file 13567_2019_642_MOESM10_ESM.doc]

**Additional file 10: miRNAs and their targets enrichments in different developmental stages in male and female.**

| **Stage** | **Gender** | **Enrichments** | **miRNAs** | **Comment/ Number of miRNAs** |
| --- | --- | --- | --- | --- |
| **Pairing stage**  **(cluster 1)** | Female | signal transduction by protein phosphorylation | miR-36-3p, miR-3484-5p, miR-219-5p, miR-2b-3p, miR-133, miR-2d-5p, miR-124-3p, miR-3489, miR-3507 | These 15 miRNAs and 3 enrichments might play a key role in the stimulation or attraction of female-male pairing, communication and immune response in parasite-host during pairing stage. |
|  |  | leukocyte proliferation | miR-219-5p, miR-124-3p, miR-71b-5p, miR-2a-5p, miR-36-3p |  |
|  |  | regulation of chemotaxis | miR-2b-3p, miR-2c-3p, miR-1, miR-2a-5p, miR-2d-3p, miR-36-3p |  |
|  |  | positive regulation of epithelial to mesenchymal transition | miR-3484-5p, miR-36-3p, miR-1, miR-3489 | 4 miRNAs |
|  |  | cellular macromolecule localization | miR-3484-5p, miR-133, miR-31-5p, miR-124-3p, let-7, miR-1, miR-3480-5p, miR-219-5p, miR-71a | 9 miRNAs |
|  |  | cell junction organization | miR-1, miR-3480-5p, miR-124-3p, miR-219-5p, miR-2a-5p, miR-7-5p, miR-133 | 7 miRNAs |
|  |  | regulation of biosynthetic process | miR-3484-5p, miR-31-5p, miR-133, miR-124-3p, miR-1, let-7, miR-219-5p, miR-2b-3p, miR-36-3p, miR-71b-5p, miR-2b-5p, miR-3507, miR-3480-5p, miR-71a, miR-2a-5p, miR-36-5p | 16 miRNAs |
|  |  | extracellular structure organization | miR-36-3p, miR-3484-5p, miR-2c-3p, miR-2a-5p, miR-219-3p, miR-7-5p, miR-133, miR-1 | 8 miRNAs |
|  |  | maintenance of protein location | miR-36-3p, miR-124-3p, miR-2d-5p, miR-31-5p, miR-133, let-7 | 6 miRNAs |
|  |  | maintenance of location in cell | miR-36-3p, miR-124-3p, miR-2d-5p, miR-31-5p, miR-133, let-7 | 6 miRNAs |
|  |  | multicellular organism metabolic process | miR-36-3p, miR-2c-3p, miR-219-3p, miR-7-5p | 4 miRNAs |
|  | Male | sex differentiation | miR-2d-5p, miR-3489, miR-3490, miR-3485-5p, miR-3504, miR-3500, miR-3479-5p | These 11 miRNAs might be involved in male sexual development regulation at the early paring stage |
|  |  | meiotic nuclear division | miR-3494, miR-2d-3p, miR-3489, miR-3485-5p, miR-3490, miR-124-5p, miR-36-5p |  |
|  |  | regulation of symbiosis, encompassing mutualism through parasitism | miR-3485-5p, miR-3504, miR-3490 | 3 miRNAs |
| **Developmental and mature stage**  **(cluster 2)** | Female | compound metabolic process | miR-3479-5p, miR-190-3p, miR-2a-3p, miR-3482-3p, miR-3483-3p, miR-3503, let-7, miR-124-5p, miR-7-3p, miR-2c-3p, miR-3494, miR-10-5p, miR-3498, miR-3501, miR-310 | 15 miRNAs |
|  |  | response to BMP | miR-36-3p, miR-2a-3p, miR-2c-5p, miR-3483-3p, miR-3488 | 5 miRNAs |
|  |  | female pregnancy | miR-3494, miR-3482-3p, miR-3479-5p, miR-3482-5p, miR-3503 | 5 miRNAs |
|  |  | spermatid differentiation | miR-3503, miR-3494 | 2 miRNAs |
|  |  | regulation of meiotic cell cycle | miR-3494, miR-124-5p | 2 miRNAs |
|  |  | maternal process involved in female pregnancy | miR-3494, miR-3482-3p | 2 miRNAs |
|  | Male | regulation of biosynthetic process | miR-3496, miR-3490, miR-133, miR-124-3p, miR-7-5p, miR-1, miR-190-5p, miR-3505, miR-219-5p, let-7, miR-3487 | 11 miRNAs |
|  |  | regulation of compound metabolic process | let-7, miR-124-3p, miR-3496, miR-1, miR-3490, miR-133, miR-7-5p, miR-3487, miR-190-5p, miR-3505, miR-219-5p | 11 miRNAs |
|  |  | regulation of immune effector process | miR-133, miR-219-5p, miR-124-3p, miR-3487 | 4 miRNAs |
|  |  | positive regulation of cell growth | miR-2c-3p, let-7, miR-7-5p, miR-133, miR-3488 | 5 miRNAs |
| **Egg production stage**  **(cluster 3)**  **Egg production stage**  **(cluster 3)** | Female | macromolecule metabolic process | miR-3490, miR-3491, miR-3487, miR-3481-5p, miR-3502, miR-7-5p, miR-36-3p, miR-8185, miR-3496, miR-3483-5p, miR-3485-5p, let-7, miR-3504, miR-3493, miR-3500, miR-3505, miR-3497, miR-3506, miR-3489, miR-3481-3p, miR-3485-3p, miR-2b-5p, miR-3499, miR-3488, miR-125a, miR-125b, bantam, miR-3486-3p, miR-190-5p, miR-3492, miR-36-5p, miR-3495 | 32 miRNAs |
|  |  | nerve development | miR-3490, miR-3483-5p, miR-3504 | 4 miRNAs might play specific role during interplay in female-male and female specialized function of egg production. |
|  |  | chemosensory behavior | miR-3483-5p, miR-3500 |  |
|  |  | biosynthetic and metabolic process | miR-8185, miR-3490, miR-3491, miR-3496, miR-3481-5p, miR-3504, miR-3505, miR-3487, miR-3483-5p, miR-7-5p, miR-36-3p, let-7, miR-3485-5p, miR-3492, miR-3500, miR-3493, miR-190-5p, miR-3489, miR-36-5p, miR-2b-5p, miR-3495, miR-3502, miR-3488, miR-3497, miR-3486-3p, bantam, miR-3485-3p, miR-133, miR-2a-3p, miR-3499, miR-125a, miR-125b, miR-3506, miR-3481-3p, | 34 miRNAs |
|  |  | cellular lipid metabolic process | miR-3490, miR-3485-5p, miR-3504, miR-7-5p, miR-2b-5p, miR-3502, miR-3493, miR-3487, bantam, miR-8185 | 10 miRNAs |
|  | Male | cellular macromolecule metabolic process | miR-36-3p, miR-3484-5p, miR-3482-3p, miR-31-5p, miR-3483-5p, bantam, miR-8185, miR-3480-5p, miR-3503, miR-125b, miR-2d-5p, miR-2b-5p, miR-2a-5p, miR-71a, miR-10-5p, miR-31-3p, miR-3481-3p, miR-3507, miR-3486-3p, miR-3502 | 20 miRNAs |
|  |  | positive regulation of epithelial to mesenchymal transition | miR-3484-5p, miR-36-3p, miR-3489 | 6 miRNAs might play specific role during interplay in female-male and female specialized function of egg production. |
|  |  | body morphogenesis | miR-3480-5p, miR-31-3p, miR-2a-5p |  |
