# Supplementary material for: Comprehensive analysis of miRNA profiles reveals the role of Schistosoma japonicum miRNAs at different developmental stages
Source: Vet Res. 2019 Apr 4;50:23. doi: 10.1186/s13567-019-0642-2 (PMC6449929; doi:10.1186/s13567-019-0642-2)
Supplement: Supplementary file 14 — Additional file 14. MiRNAs regulated different transferase activities in female and male. [file 13567_2019_642_MOESM14_ESM.docx]

**Additional file 14:** **MiRNAs regulated different transferase activity in female and male.**

| **cluster** | **Subcategories group** | **miRNAs related** | **Specific subcategories group** | **Specific miRNAs related** |
| --- | --- | --- | --- | --- |
| F-cluster 3 | Total: 22  glycine hydroxymethyl transferase, unc51-like kinase  Alanine aminotransferase, histone-lysine N-methyltransferase,  classical protein kinase C, phosphatidylinositol-4,5-bisphosphate 3-kinase  alpha-1,3-glucosyltransferase, serine/threonine kinase  histone acetyltransferase, ethanolamine-phosphate cytidylyl transferase, Polynucleotidyl transferase, serine/threonine protein kinase, phosphatidylinositol 4-kinase, thiamine pyrophospho kinase, protein phosphatase, dimethyladenosine transferase, O-methyltransferase, nicotinamide phosphoribosyl transferase, tRNA-ribosyl transferase, mitogen-activated protein kinase kinase, Diphthine synthase, Tau-tubulin kinase | miR-8185, miR-3485-5p, miR-3490, miR-3505, miR-3483-5p, miR-36-3p, miR-3492, miR-3496, miR-3487, miR-3500, miR-3493, miR-190-5p  miR-3506, miR-3489, miR-3504, miR-2b-5p, miR-3502, miR-7-5p  miR-3488, miR-3486-3p, miR-3481-3p, miR-3485-3p, miR-2a-3p, miR-3497, miR-3499  miR-2c-5p, miR-1 | classical protein kinase C, phosphatidylinositol-4,5-bisphosphate 3-kinase, alpha-1,3-glucosyltransferase,  ethanolamine-phosphate cytidylyl transferase,  Polynucleotidyl transferase, phosphatidylinositol 4-kinase, thiamine pyrophospho kinase, protein phosphatase,  dimethyladenosine transferase,  nicotinamide phosphoribosyl transferase,  tRNA-ribosyl transferase,  mitogen-activated protein kinase kinase,  Diphthine synthase, Tau-tubulin kinase. | miR-190-5p，miR-7-5p  miR-1, miR-3487, miR-3505, miR-3496, miR-3488, |
| M-cluster 1 | Total: 16  serine/threonine-protein kinase, unc51-like kinase,  RNA methyltransferase-like protein 1B, O-sialoglycoprotein endopeptidase, nicotinamide phosphoribosyl transferase,  methyltransferase UPF0383, Alanine aminotransferase 2,  homocysteine S-methyltransferase, alpha-1,2-mannosyltransferase,  serine/threonine-protein kinase Chk2, queuine tRNA-ribosyl transferase, Histone-lysine N-methyltransferase SETD8,  rRNA 2'-O-methyltransferase, fibrillarin 1, alpha-1,3-glucosyltransferase, histone acetyltransferase, thiamine pyrophospho kinase. | miR-3485-5p, miR-3497, miR-3479-5p, miR-190-3p, miR-71b-5p, miR-3504, miR-2a-3p  miR-3499, miR-3490, miR-2d-3p  miR-36-3p, miR-3492, miR-36-5p  let-7, miR-3481-3p, miR-2d-5p  miR-3489, miR-310, miR-3500  miR-2c-5p, miR-3493, miR-2c-3p  miR-3506, miR-3494, miR-124-5p  miR-2b-3p, miR-3502, miR-3485-3p | RNA methyltransferase-like protein 1B, O-sialoglyco protein endopeptidase, methyltransferase UPF0383, Alanine aminotransferase 2, homocysteine S-methyltransferase, alpha-1,2-mannosyl transferase, serine/threonine-protein kinase Chk2, queuine tRNA-ribosyl transferase, Histone-lysine N-methyltransferase SETD8, rRNA 2'-O-methyltransferase, fibrillarin 1 | miR-3479-5p, miR-190-3p,  miR-71b-5p, miR-2d-3p,  miR-36-5p, let-7, miR-2d-5p,  miR-310, miR-2c-3p,  miR-3494, miR-124-5p,  miR-2b-3p |
| M-cluster 3 | Total: 9  glycine hydroxymethyl transferase, tRNA (guanine-N2-)-methyltransferase, Glycoprotein-N-acetylgalactosamine, 3-beta-galactosyltransferase, palmitoyl transferase, ZDHHC5, ethanolamine-phosphate cytidylyl transferase, Histone-lysine N-methyltransferase SETD8, O-methyltransferase, O-phosphoseryl-tRNA(Sec) selenium transferase, nicotinamide phosphoribosyl transferase. | miR-3483-5p, miR-8185, miR-3484-5p, miR-3482-3p, miR-36-3p, miR-3480-5p, miR-3481-3p, miR-2c-5p, miR-31-5p  miR-3503, miR-2a-5p, miR-10-5p  miR-71a, miR-2b-5p, miR-3507  miR-3486-3p, miR-3502, miR-31-3p | tRNA (guanine-N2-)-methyltransferase,  Glycoprotein-N-acetylgalactosamine 3-beta-galactosyltransferase,  Palmitoyl transferase, ZDHHC5, O-phosphoseryl-tRNA (Sec), selenium transferase. | miR-3484-5p，miR-3482-3p,  miR-3480-5p,  miR-31-5p, miR-3503  miR-2a-5p, miR-10-5p  miR-71a, miR-3507, miR-31-3p |
